# Temporal profiling of primary metabolites under chilling stress and its association with seedling chilling tolerance of rice (*Oryza sativa* L.)

**DOI:** 10.1186/1939-8433-6-23

**Published:** 2013-10-05

**Authors:** Xiu-Qin Zhao, Wen-Sheng Wang, Fan Zhang, Ting Zhang, Wen Zhao, Bin-Ying Fu, Zhi-Kang Li

**Affiliations:** Institute of Crop Sciences / National Key Facility for Crop Gene Resources and Genetic Improvement, Chinese Academy of Agricultural Sciences, Beijing, 100081 China; Institute of Vegetables and Flowers, Chinese Academy of Agricultural Sciences, Beijing, 100081 China

**Keywords:** Primary metabolites, Seedling chilling tolerance, Transcriptomic responses, Rice

## Abstract

**Background:**

Chilling stress is a major factor limiting rice production. Rice genotypes differ greatly in their seedling chilling tolerance (CT), which is known to involve differential expression of large numbers of genes and proteins. To further understand the metabolomic responses of rice to chilling stress, profiles of the 106 primary metabolites of a CT *japonica* variety, Lijiangxintuanhegu (LTH) and a chilling sensitive *indica* line, IR29, were investigated under a time-series of chilling stress and non-stress control conditions at the seedling stage.

**Results:**

We identified 106 primary metabolites that were temporally and genotype-dependently regulated in LTH and IR29 under the time-series chilling stress and subsequent recovery. Three major groups of primary metabolites, amino acids (AAs), organic acids (OAs) and sugars, showed distinct change patterns in both genotypes in response to the chilling stress: a more general accumulation of most AAs, more dramatic decreased levels of most OAs, and greatly reduced levels for most sugars at early time points of stress but increased levels of specific sugars at the later time points of stress. Compared to IR29, LTH had more metabolites showing chilling induced changes, greater levels of these metabolomic changes and a greater ability to recover after stress, implying that LTH used a positive energy-saving strategy against chilling stress. During subsequent recovery, more metabolites were significantly and exclusively up-regulated in LTH, indicating their positive role in chilling tolerance. A comparative analysis of these metabolites data and differentially expressed genes data allowed identification of 7 AAs and related genes that were both chilling responsive and contributed greatly to the CT of LTH.

**Conclusions:**

The metabolomic responses of rice to chilling stress at the seedling stage were dynamic and involved large numbers of the metabolites. The chilling induced changes of three major groups of metabolites, AAs, OAs and sugars, in rice were well coordinated. The high level seedling CT of LTH was apparently attributed to its increased levels of most AAs and reduced energy consumption that resulted in increased glycolysis and strong resilience on recovery. The results of this study extend our understanding of molecular mechanisms of chilling stress tolerance in rice.

**Electronic supplementary material:**

The online version of this article (doi:10.1186/1939-8433-6-23) contains supplementary material, which is available to authorized users.

## Background

Cultivated rice (*Oryza sativa* L), originated in tropical areas, is susceptible to cold (De Datta, 1981). Low temperature (LT) is accordingly one of the major constraints on rice production and productivity in the temperate rice-growing countries and high-altitude tropical areas (Kanada 1974; Shimono et al. 2002; Andaya and Tai 2006; Oliver et al. 2007; Suzuki et al. 2008; Wang et al. 2013). During a normal rice crop season, LT affects germination and seedling vigor, delays plant development and heading at the early developmental stages (Lee et al. 1996; Humphreys et al. 1996; Oliver et al. 2007; Cheng et al. 2007), and causes pollen sterility and severe yield losses at the reproductive stage (Angus and Lewin 1991; Jacobs and Pearson 1999; Oliver et al. 2007). Differentiation of *O*. *sativa* into two major subspecies, *O*. *sativa indica* and *O*. *sativa japonica*, is well known to be associated with their general geographic distributions and adaptations to the tropical and low altitude areas (*indicas*) and the temperate and high altitude environments (*japonicas*). As a result, most *indica* varieties are more vulnerable to chilling than those of *japonicas* at both the seedling and reproductive stages (Lee et al. 1993; Cheng et al. 2007).

Plants respond to cold stress by altering gene, protein, and metabolite expression and cell membrane lipid composition (Levitt 1972; Gilmour et al. 2000; Shinozaki and Dennis, 2003). As revealed by comprehensive transcriptomic and proteomic analyses, many genes and functional proteins are involved in rice response to chilling (Seki et al. 2001; Fowler and Thomashow 2002; Kreps et al. 2002; Sakuma et al. 2002; Hashimoto and Komatsu 2007; Zhang et al. 2012a, b; Nakashima et al. 2007; Kanneganti and Gupta 2008; Ma et al. 2009; Oh et al. 2009). For example, many proteins responsible for compatible compound synthesis are involved in cold tolerance regulation in rice. These proteins include otsA and otsB, choline monooxygenase, and WFT1 and WFT2, which are involved in synthesis of trehalose, glycinebetaine, and fructan, respectively (Garg et al. 2002; Shirasawa et al. 2006; Kawakami et al. 2008).

In contrast to the strong research focus on genes and proteins associated with cold tolerance, a handful of studies have examined plant responses to chilling stress at the metabolomic level. In *Arabidopsis thaliana*, the acclimation abilities of different Arabidopsis ectotypes under cold stress was reported to be dependent on and correlated with the massive metabolomic changes (Cook et al. 2004; Davey et al. 2009), and combinations of specific metabolites are predictative of leaf freezing tolerance and of heterosis in freezing tolerance (Hannah et al. 2006; Korn et al. 2010). For example, many metabolites such as sugars, amino acids (AAs) and polyamines were highly induced under cold stress in Arabidopsis and other plant species (Kaplan et al. 2004; Guy et al. 2008). Also, carbohydrate metabolism has been shown to play a crucial role in cold stress response in plants (Nägele et al. 2012) and the accumulation of a few metabolites including glucose, fructose, sucrose and raffinose were found to be significantly correlated with CT (Zuther et al. 2004; Maruyama et al. 2009). Meanwhile, a number of AAs were found to increase in response to cold stress in wheat (Naidu et al. 1991). Proline has been shown to function as both an osmotic agent and a radical scavenger in plants under low temperature stress (Hayat et al. 2012; Kavi Kishor and Sreenivasulu, 2013). There is ample evidence that many metabolites are involved in plant responses to low temperature stress, though their reconfiguration under stress is complex and involves multiple molecular pathways (Guy et al. 2008).

As part of our overall effort to dissect molecular mechanisms of CT of rice, we present here our effort in quantifying differential metabolite responses to chilling stress of two rice genotypes differing greatly in CT. Our results provide some insights into the complexity of the metabolomic responses of rice to chilling stress at the seedling stage and their possible contributions to the seedling CT of rice.

## Results

### The genotypic difference in cell membrane injury caused by the chilling treatments

**Figure 1:**
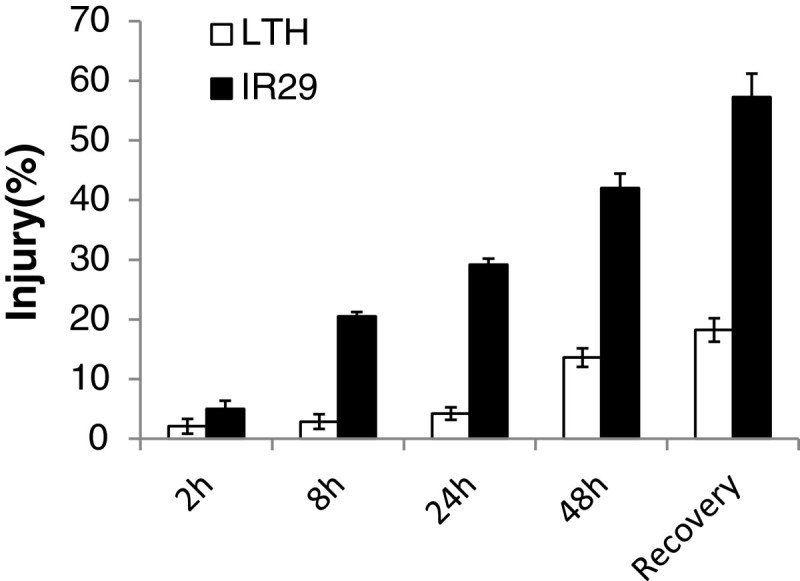
**Mean genotypic differences in cell membrane injury between LTH and IR29 under the 4°C chilling stress and non-stress control for 2, 8, 24, and 48 h and subsequent recovery for 24 h.** Vertical bars indicate the standard errors.

Figure 1 shows the differences in cell membrane injury between LTH and IR29 measured as their relative electrolyte leakages under the chilling treatments at 4°C for 2 h, 8 h, 24 h, and 48 h, and upon recovery for 24 h (R-24 h) after the chilling treatment. Significant difference in cell membrane injury was observed between LTH (2.1%) and IR29 (5.0%) at 2 h under the 4°C chilling treatment. This difference increased dramatically to 17.6% at 8 h of the chilling treatment, and reached ~25% at 24 h and 48 h of the chilling treatment. At the R-24h after the chilling treatment, cell membrane injury was 18.2% in LTH and 57.3% in IR29. These results were consistent with our previous result (Zhang et al. 2012b) that LTH has much better seedling CT than IR29.

### Metabolite profiling of LTH and IR29 under control and chilling stress treatments

Additional file 1: Table S1 shows the GC-MS measurements of 106 primary metabolites from leaf samples of LTH and IR29 under control and 4°C chilling stress conditions for 2 h, 8 h, 24 h, and 48 h, and R-24 h. These metabolites included 15 AAs, 31 organic acids (OAs), 38 sugars, 9 esters, 4 polyamines, and 9 other small molecular components (SMCs). ANOVA results indicate that of the 106 measured metabolites, 82 showed significant differences between LTH and IR29, which, on average, explained 18.1% of the total phenotypic variation of the measured metabolites (Additional file 2: Table S2). The chilling treatment caused significant changes in 76 measured metabolites, which, on average, explained 18.7% of the total phenotypic variation in these metabolites. The measurement timing had large effects on all measured metabolites except for tryptophan, octadecanoic acid, phosphoric acid and erythritol, which, on average, explained 34.0% of the total phenotypic variation of the measured metabolites. Genotype by measurement timing interaction was significant for 88 metabolites and explained an average of 18.6% of the total phenotypic variation of the metabolites (Additional file 2: Table S2).

**Figure 2:**
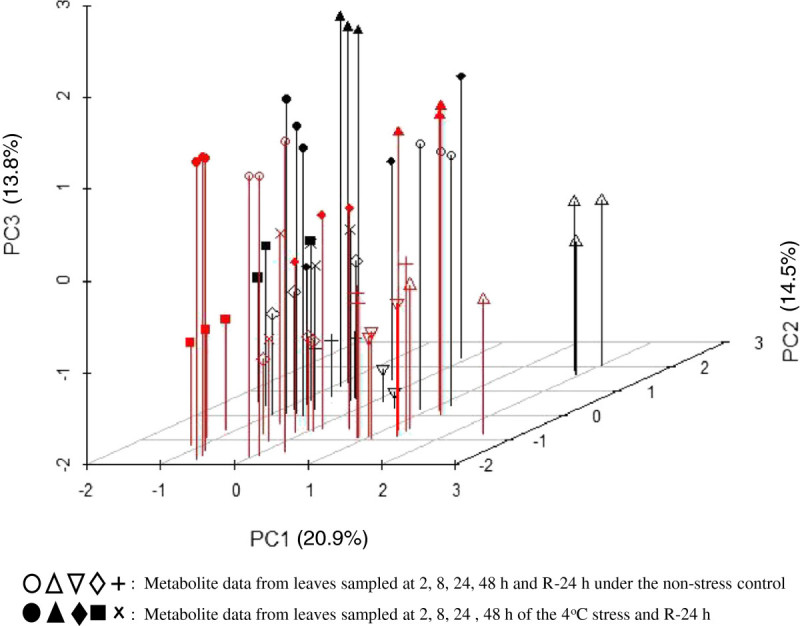
**The plot of the first three principal components of the leaf metabolites in two rice genotypes, LTH and IR29, sampled in three biological replicates at the time points of 2, 8, 24, and 48 h after the 4°C chilling stress and 24 h after recovery (R).** The red color and black one indicated IR29 and LTH.

To get an overall picture of the metabolite profiling of the two rice genotypes in response to the chilling treatments, the measured metabolite data were subjected to a principal component (PC) analysis. As shown in Figure 2, PC1 accounted for 20.9% of the total detected metabolite variance and clearly separated the chilling and control treatments on the negative (decreased) and positive (increased) sides of *X* axis of the PC plot. Metabolites with heavy negative loadings (chilling responsive) on this PC included 9 of the 15 AAs, 1 polyamine (triethanolamine), 1 ester (threonic acid-1,4-lactone) plus 1 SMC (uracil), while 25 of the 31 OAs, 27 of the 38 sugars, 7 of the 9 esters, 6 other SMCs plus one AA (glycine) had heavy positive loadings on this PC (Additional file 3: Table S3).

PC2 accounted for 14.5% of the total detected metabolite variance, which clearly separated the two genotypes, LTH on the positive side and IR29 on the negative side, of *Y* axis of the PC plot. Metabolites with heavy positive loadings on this PC included 8 of the 15 AAs, 7 of the 31 OAs, 16 of the 38 sugars, 2 of the 4 polyamines, 5 of the 9 esters plus 6 other SMCs, while 2 of the 15 AAs, 17 of the 31 OAs, 12 of the 38 sugars plus 2 other SMCs contributed negatively to this PC (Additional file 3: Table S3). The PC3 explained 13.8% of the total metabolite variance and represented the temporal variation of the sampling time points with early sampling at 2 h and 8 h on the positive side and the late sampling at 48 h and R-24 h on negative side (Figure 2). Metabolites with heavy positive loadings on this PC included 13 of the 15 AAs (except for alanine and leucine), 11 of the 31 OAs, 18 of the 38 sugars, 1 of the 9 esters plus 5 other SMCs, while 5 of the 31 OAs, 9 of the 38 sugars, 2 polyamines plus 2 esters contributed negatively to this PC (Additional file 3: Table S3). The PCA result also implicates that those metabolites with heavy negative loadings on PC1 and positive loading on PC2 in the Figure 2 were the most important ones that contributed to the difference in the seedling CT between LTH and IR29, which included only 7 AAs: proline, valine, leucine, tryptophan, threonine, serine and tyrosine (Additional file 3: Table S3).

### Time-dependent metabolite responses of LTH and IR29 under chilling stress

**Figure 3:**
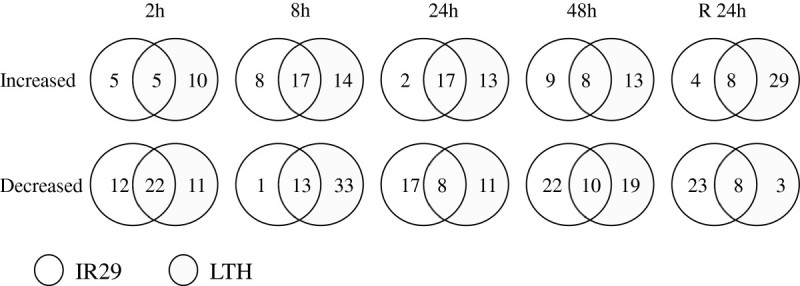
**Venn diagrams showing coordinated significant (p ≤ 0.05) increases and decreases between two rice genotypes, LTH and IR29, for a set of 106 identified metabolites measured at the time points of 2, 8, 24, and 48 h of the 4°C chilling stress and 24 h after recovery.**

When the level of a metabolite showing a significant change (p ≤ 0.05 based on ANOVA) relative to its respective control was defined as differentially regulated, all measured metabolites except for octadecanoic acid, galacturonic acid and monostearoylglycerol, were differentially regulated in both genotypes at one or more time points during the chilling stress (Table 1). Figure 3 shows the number of genotype-specific and commonly regulated metabolites based on the comparative analysis of the differentially regulated metabolites in LTH and IR29. Overall, the number of the differentially regulated metabolites was 65, 86, 68, 81 and 73 at 2 h, 8 h, 24 h, 48 h and R-24 h of the chilling treatment. The number of metabolites showing significantly increased levels was smaller than that of showing significantly decreased levels at all time points except at the R-24 h. Compared to IR29, LTH had more metabolites showing significantly changed levels and a greater extent of most of these changes under the chilling treatment (Figure 3, Table 1, Additional file 1: Table S1). Throughout the chilling treatment, threonine, isoleucine and valine were the only three metabolites that commonly increased in both genotypes, while quinic acid was commonly decreased significantly (p ≤ 0.05) in both genotypes (Table 1).

**Table 1:** **The metabolites changes in seedlings of IR29 and LTH under different time points of chilling stress and subsequent recovery conditions**

	2 h		8 h		24 h		48 h		R-24 h	
	IR29	LTH	IR29	LTH	IR29	LTH	IR29	LTH	IR29	LTH
**Amino acids**										
Alanine	-1.2	**1.2**	1.3	**1.7**	**2.2**	**1.8**	**1.6**	**2.0**	1.2	1.1
Aspartic acid	1.1	1.4	**2.6**	**2.1**	**1.9**	**5.1**	-1.1	1.3	- *1* . *7*	**1.4**
Cysteine	- *1* . *3*	- *1* . *7*	**1.9**	**4.8**	**4.8**	-1.2	**3.2**	1.1	**6.7**	1.4
Glutamic acid	-1.2	**1.4**	**2.9**	1.5	- *1* . *6*	**4.4**	- *1* . *8*	- *1* . *4*	**2.0**	1.2
Glycine	1.1	-1.1	-1.3	**1.6**	- *2* . *0*	1.0	- *1* . *6*	**1.4**	-1.1	**1.7**
Isoleucine	**1.6**	**3.1**	**4.0**	**18.0**	**7.8**	**4.5**	**3.4**	**3.6**	**2.6**	**1.8**
Leucine	1.2	**1.9**	1.5	**2.8**	**2.6**	**2.6**	**1.5**	**1.9**	1.3	-1.1
Lysine	1.0	**1.5**	**2.5**	**1.9**	**2.0**	**2.0**	1.3	**1.5**	1.4	**1.3**
Phe	**1.4**	1.3	**2.1**	**1.5**	**1.6**	**2.9**	1.1	**1.6**	1.3	**1.8**
Proline	**1.4**	**1**.**8**	1.5	**3**.**5**	**1**.**9**	**4**.**4**	**1**.**3**	**1**.**8**	-1.2	1.1
Serine	1.2	**1**.**6**	**2**.**7**	**4**.**4**	**1**.**8**	**2**.**3**	**1**.**3**	1.1	1.0	1.1
Threonine	**1**.**4**	**1**.**7**	**6**.**7**	**3**.**5**	**2**.**6**	**4**.**2**	**1**.**8**	**1**.**8**	1.1	**1**.**3**
Tryptophan	1.3	**2**.**4**	**2**.**7**	**2**.**6**	**2**.**4**	**3**.**9**	1.1	**2**.**3**	1.2	**2**.**7**
Tyrosine	1.2	1.4	**2**.**1**	**2**.**2**	**2**.**0**	**2**.**6**	1.1	**2**.**1**	-1.2	**1**.**4**
Valine	**1**.**6**	**2**.**7**	**3**.**3**	**9**.**7**	**3**.**8**	**6**.**8**	**2**.**4**	**2**.**8**	1.4	**1**.**8**
**Organic acids**										
Heptadecoic acid	-1.1	-1.1	-1.1	- *2* . *0*	1.0	1.1	-1.1	-1.0	-1.1	-1.1
Octadecanoic acid	1.0	-1.2	1.0	-1.0	1.1	1.2	-1.1	1.0	1.3	1.0
Oleic acid	-1.1	- *1* . *3*	-1.2	-1.2	- *1* . *2*	-1.2	- *1* . *6*	- *1* . *7*	1.0	- *1* . *6*
Palmic acid	-1.1	-1.2	-1.1	-1.2	1.0	1.2	-1.2	-1.1	-1.1	1.0
Quinic acid	- *2* . *4*	- *4* . *4*	- *4* . *6*	- *8* . *3*	- *3* . *1*	- *6* . *7*	- *5* . *3*	- *1* . *7*	- *1* . *8*	-1.4
Succinic acid	- *1* . *4*	-1.2	-1.3	- *1* . *4*	-1.3	- *2* . *0*	- *1* . *4*	- *2* . *1*	1.0	1.1
Aminobutyric acid, GABA	-1.1	-1.2	1.0	- *1* . *5*	-1.2	-1.1	- *2* . *4*	1.0	- *1* . *6*	-1.2
Benzoic acid	-1.1	- *1* . *4*	-1.0	-1.0	1.1	-1.1	-1.2	-1.2	-1.3	**1**.**7**
Cinnamic acid, 4-hydroxy	-1.1	-1.1	-1.1	- *1* . *2*	-1.1	-1.3	-1.3	- *1* . *9*	- *1* . *8*	-1.2
Eicosanoic acid	- *1* . *5*	- *1* . *9*	-1.2	- *9* . *1*	- *2* . *0*	- *4* . *0*	- *2* . *0*	- *2* . *0*	- *4* . *5*	-1.5
Oxalic acid	**11**.**9**	- *2* . *6*	- *1* . *8*	- *3* . *9*	**1**.**3**	**1**.**8**	1.5	**1**.**7**	- *8* . *9*	- *4* . *1*
Phosphoric acid	- *1* . *3*	-1.2	-1.0	- *2* . *3*	-1.2	1.1	-1.1	-1.6	- *1* . *5*	-1.2
Pyruvic acid	1.0	1.0	-1.2	**1**.**3**	- *1* . *2*	-1.1	-1.1	- *1* . *5*	-1.5	1.2
Shikimic acid	1.2	-1.1	- *12* . *5*	- *10* . *0*	- *7* . *1*	- *4* . *0*	- *1* . *4*	**1**.**4**	- *2* . *0*	- *2* . *9*
Sinapic acid	-1.1	-1.4	1.2	- *2* . *9*	1.0	- *2* . *3*	-1.0	- *3* . *3*	- *4* . *8*	- *3* . *6*
Citric acid	- *1* . *3*	1.1	-1.1	1.0	- *1* . *9*	- *2* . *3*	- *2* . *0*	-1.0	- *1* . *5*	1.1
Erythronic acid	- *1* . *5*	-1.5	-1.5	- *2* . *2*	-1.5	-1.2	-1.2	- *1* . *6*	- *1* . *4*	**1**.**3**
Fumaric acid	- *1* . *3*	- *1* . *9*	- *1* . *8*	- *2* . *3*	1.1	- *2* . *1*	**2**.**2**	- *1* . *7*	-1.0	1.1
Isocitric acid	1.1	**1**.**7**	-1.1	**5**.**8**	-1.3	-1.2	- *1* . *4*	1.1	- *3* . *9*	- *2* . *3*
Malic acid	- *2* . *1*	- *2* . *3*	- *3* . *1*	- *3* . *7*	-1.2	**1**.**6**	- *1* . *6*	-1.2	- *1* . *7*	-1.1
Salicylic acid	1.1	-1.1	**1**.**3**	**1**.**8**	-1.0	- *1* . *4*	-1.4	- *2* . *2*	-1.2	1.1
Calendic acid	1.0	-1.1	1.3	- *2* . *2*	1.0	1.1	-1.5	- *1* . *9*	- *1* . *5*	1.2
Ascorbic acid	-1.5	1.1	**1**.**8**	**2**.**3**	**2**.**3**	**1**.**6**	1.2	1.3	- *1* . *7*	1.1
Ferulaic acid	1.1	-1.0	1.1	- *1* . *5*	- *1* . *3*	-1.2	- *1* . *7*	- *2* . *1*	- *2* . *2*	- *1* . *8*
Galacturonic acid	-1.2	1.5	1.2	-1.0	1.1	-1.2	-1.1	-1.3	-1.7	1.1
3-deoxy-arabino-hexaric acid	-1.2	- *1* . *4*	-1.1	- *2* . *2*	1.1	1.0	1.1	- *1* . *6*	-1.5	**1**.**7**
Glucopyranuronic acid	**2**.**9**	**1**.**6**	1.1	-1.5	**1**.**7**	**1**.**5**	**1**.**6**	**1**.**4**	- *9* . *4*	-1.2
Gluconic acid	-1.1	-1.1	1.2	-1.2	-1.2	- *1* . *9*	-1.3	-1.2	- *2* . *5*	1.1
Gluconic acid-6-p	- *2* . *5*	-1.0	-1.2	**1**.**5**	-1.0	- *2* . *6*	- *3* . *9*	- *1* . *8*	1.0	-1.1
Saccharic acid	1.2	-1.5	-1.2	-1.6	-1.0	-1.0	**2**.**1**	1.5	1.2	**1**.**7**
Galactonic acid	1.0	1.1	-1.2	**1**.**3**	-1.0	1.0	1.1	-1.1	- *1* . *8*	**1**.**7**
**Sugars**										
2-o-glycerol-beta-d-galalctopyranoside	-1.3	1.0	1.2	1.0	-1.1	1.3	-1.0	- *3* . *5*	-1.4	**1**.**8**
Benzyl glucopyranoside	-1.1	1.1	**1**.**7**	**1**.**3**	- *2* . *4*	1.1	**2**.**0**	**1**.**5**	**1**.**7**	1.2
Galactinol	-1.0	-1.0	**1**.**4**	**1**.**4**	**1**.**7**	**1**.**5**	**1**.**7**	-1.1	- *2* . *1*	- *2* . *2*
Methyl-beta-d-mannopyranoside	1.2	- *2* . *6*	1.0	- *3* . *1*	-1.2	-1.2	1.2	**4**.**3**	-1.2	**6**.**0**
Ethylglucopyranoside	- *1* . *4*	- *1* . *3*	**1**.**9**	**1**.**8**	-1.2	- *1* . *8*	**1**.**6**	-1.2	- *2* . *1*	1.4
Arabinose	-1.3	-1.1	1.2	**1**.**3**	-1.0	1.2	-1.2	- *1* . *5*	**2**.**4**	**2**.**3**
Cellobiose	-1.1	-1.1	**2**.**1**	-1.3	-1.0	**1**.**6**	- *1* . *6*	- *1* . *6*	-1.1	-1.1
Fructofuranose	- *1* . *3*	-1.4	1.4	- *1* . *6*	-1.2	**1**.**5**	- *1* . *5*	-1.2	1.0	1.1
Fructose	- *1* . *4*	- *1* . *9*	-1.4	- *1* . *9*	- *1* . *3*	-1.1	-1.3	-1.1	-1.2	-1.1
Glucopyranose	- *1* . *3*	- *1* . *9*	-1.2	- *1* . *7*	-1.1	-1.1	-1.3	1.1	-1.1	-1.1
Glucose	- *1* . *3*	- *1* . *7*	1.5	- *1* . *6*	- *1* . *4*	-1.3	- *1* . *5*	1.0	-1.1	1.1
Glucose-6-p	- *2* . *0*	- *2* . *0*	- *1* . *7*	- *2* . *0*	- *1* . *3*	**1**.**9**	1.3	**2**.**3**	-1.2	1.0
Mannitose	- *1* . *4*	- *1* . *8*	- *1* . *6*	- *1* . *9*	- *1* . *3*	-1.1	-1.4	1.1	-1.3	-1.1
Melibiose	1.1	-1.2	1.4	-1.1	-1.1	**1**.**8**	-1.2	-1.1	-1.5	-1.1
Rhamnose	- *1* . *3*	1.0	1.3	- *1* . *2*	1.1	1.3	-1.2	-1.0	1.3	-1.1
Ribose	- *1* . *4*	1.0	1.3	**1**.**7**	1.0	-1.3	- *2* . *3*	-1.2	**1**.**7**	**4**.**8**
Sorbinose	- *1* . *2*	- *2* . *0*	-1.1	- *1* . *3*	-1.1	-1.1	- *1* . *4*	1.0	1.0	1.1
Fucose	-1.2	**1**.**3**	1.2	**1**.**6**	-1.3	-1.4	-1.6	-1.0	1.2	**1**.**9**
Galactose	- *1* . *3*	- *1* . *6*	1.3	- *2* . *3*	1.3	-1.3	- *1* . *5*	-1.2	**1**.**6**	**2**.**3**
Lactose	- *4* . *6*	- *33* . *3*	- *4* . *0*	- *33* . *3*	- *2* . *4*	-1.2	- *2* . *0*	**12**.**9**	- *2* . *2*	-1.1
Maltose	- *1* . *3*	-1.2	-1.1	- *1* . *6*	- *1* . *3*	- *1* . *5*	- *1* . *5*	-1.0	-1.4	**2**.**0**
Melicitose	- *2* . *0*	- *5* . *0*	-1.2	- *1* . *5*	- *1* . *4*	**1**.**6**	-1.6	1.0	- *1* . *7*	1.0
Raffinose	- *2* . *4*	- *7* . *1*	- *2* . *3*	- *7* . *7*	- *2* . *0*	-1.3	- *2* . *9*	**3**.**1**	- *1* . *6*	-1.2
Sorbitol	1.2	1.3	1.2	-2.0	1.1	-1.4	-1.1	-1.0	1.2	**1**.**6**
Sucrose	- *5* . *3*	- *100* . *0*	- *4* . *4*	- *33* . *3*	- *2* . *4*	-1.2	- *2* . *1*	**22**.**3**	- *2* . *2*	-1.1
Trehalose	- *2* . *1*	- *1* . *4*	-1.1	- *1* . *7*	-1.3	-1.3	-1.4	- *1* . *6*	- *3* . *3*	- *1* . *6*
Turanose	- *1* . *4*	- *1* . *2*	-1.2	- *2* . *3*	1.1	-1.2	-1.0	-1.1	- *1* . *9*	1.4
Fructose-6-p	- *1* . *8*	- *1* . *9*	- *2* . *1*	-1.7	-1.1	1.3	-1.0	**1**.**7**	1.0	-1.2
Glycerol	-1.1	- *1* . *7*	1.2	- *1* . *5*	1.0	-1.2	- *1* . *5*	-1.3	1.2	**1**.**2**
Nucite	-1.1	-1.0	1.2	- *1* . *3*	1.2	1.0	-1.1	1.0	-1.2	1.1
Pentitol	**3**.**4**	- *1* . *6*	1.1	**1**.**4**	-1.2	-1.2	-1.4	-1.3	1.6	**2**.**2**
3-p-glycerol	-1.1	- *1* . *5*	-1.1	- *1* . *4*	- *1* . *5*	1.2	-1.3	- *1* . *8*	- *2* . *2*	-1.1
Galactitol	-1.1	- *1* . *3*	1.3	-1.0	-1.1	- *1* . *5*	1.1	- *1* . *7*	-1.4	**2**.**2**
Erythritol	1.0	1.0	- *1* . *8*	- *2* . *5*	1.2	- *10* . *0*	***7*** . ***7***	-1.2	1.4	1.2
Inositol-2-p	- *1* . *7*	-1.2	-1.2	- *2* . *2*	- *1* . *5*	1.2	-1.3	- *1* . *6*	-1.2	**1**.**8**
Mannitol	1.0	-1.1	1.6	**1**.**5**	1.2	**1**.**5**	1.1	1.1	1.3	**2**.**5**
Xylitol	-1.1	-1.2	**1**.**9**	**1**.**9**	-1.1	**2**.**2**	-1.1	- *1* . *3*	1.7	**4**.**4**
Sorbitol-6-p	- *3* . *2*	- *5* . *6*	- *2* . *0*	- *11* . *1*	- *7* . *1*	- *8* . *3*	- *2* . *4*	1.2	1.0	1.0
**Polyamines**										
Ethanolamine	- *1* . *3*	-1.0	1.1	-1.0	1.1	1.1	-1.2	-1.1	1.2	**1**.**2**
Putrescine	-1.4	-1.0	**1**.**7**	- *8* . *3*	**1**.**3**	1.2	- *1* . *6*	-*2*.*1*	**170**.**9**	- *1* . *7*
Triethanolamine	**1**.**3**	-1.0	**1**.**8**	1.0	-1.1	-1.1	-1.5	-1.5	**1**.**8**	**2**.**1**
Tryptamine-5-hydroxy	1.2	**1**.**9**	-1.5	- *1* . *9*	1.5	-1.1	- *2* . *6*	-1.5	- *1* . *9*	- *2* . *2*
**Esters**										
Dihexadecanoylglycerol	1.0	-1.2	**1**.**5**	-1.1	-1.1	1.0	**1**.**4**	- *1* . *4*	-1.4	-1.2
Monooctadecanoylglycerol	1.1	1.0	1.1	-1.2	1.1	1.2	-1.0	-1.0	1.4	**1**.**5**
Monostearoylglycerol	1.0	-1.2	1.1	-1.3	-1.1	1.0	-1.1	1.2	-1.0	1.1
Threonic acid-1,4-lactone	-1.1	1.1	-1.2	**1**.**6**	-1.0	1.0	1.0	-1.0	-1.1	-1.0
Gluconic acid 1,4-lactone	- *1* . *3*	- *1* . *5*	- *1* . *6*	- *2* . *6*	-1.4	-1.2	-1.4	-1.2	-1.2	1.0
Pentonic acid-1,4-lactone	-1.2	1.0	**1**.**9**	- *1* . *6*	- *1* . *6*	- *2* . *1*	- *2* . *4*	- *1* . *5*	-1.3	**1**.**4**
Monohexadecanoylglycerol	-1.2	-1.1	1.1	-1.2	1.0	1.2	1.1	1.1	**1**.**7**	**1**.**5**
Galactosylglycerol	-1.1	-1.2	1.4	- *2* . *2*	-1.1	- *1* . *7*	-1.7	- *1* . *7*	-1.2	-1.3
Digalactosylglycerol	- *1* . *4*	- *2* . *3*	**1**.**9**	- *5* . *3*	1.2	- *3* . *2*	-1.1	- *1* . *5*	1.5	- *2* . *0*
**Others**										
Cytosine	-1.3	1.4	1.4	-1.1	- *1* . *7*	1.2	-1.4	- *1* . *5*	**3**.**8**	**3**.**1**
Adenosine	1.1	-1.2	-1.1	-1.4	-1.2	-1.1	1.0	-1.1	- *1* . *9*	**1**.**5**
Allantoin	-1.1	1.2	**2**.**8**	**2**.**5**	- *1* . *4*	- *1* . *3*	**1**.**9**	-1.1	**1**.**7**	**7**.**2**
Uracil	- *1* . *5*	**1**.**7**	1.7	1.3	**2**.**2**	**2**.**9**	-1.3	-1.0	1.1	**2**.**1**
Vitamin E	**2**.**6**	- *2* . *1*	**1**.**7**	- *3* . *3*	-1.2	1.2	1.0	-1.0	- *21* . *4*	-1.5
Beta-sitosterol	-1.2	-1.0	1.1	-1.0	-1.1	**2**.**5**	- *1* . *5*	1.4	-1.4	1.0
Campesterol	-1.1	1.2	1.1	-1.2	-1.0	**2**.**1**	- *1* . *6*	1.2	-1.4	1.3
Fucosterol	-1.1	-1.4	1.1	1.1	1.1	**1**.**7**	- *2* . *2*	1.1	-1.2	1.1
Stiqmasterol	-1.2	-1.2	1.1	-1.1	1.2	**2**.**0**	- *1* . *5*	1.3	-1.3	1.1

Metabolite data obtained from leaves of IR29 and LTH at 2, 8, 24 and 48 h of the 4°C chilling treatment and 24 h after recovery were normalized to their respective untreated samples at the same growth stage. Normalized ratios less than 1.0 were inverted and multiplied by -1. Values that were increased or decreased statistically significantly (p≤0.05) are bolded or italicized-underlined, respectively.

AA *accumulation under chilling stress*: The chilling treatment resulted in generally increased levels of all 15 measured AAs in LTH and IR29 (Table 1; Additional file 1: Table S1). Of the total 120 stress vs control comparisons, 85 (71%) cases had significantly increased AA levels, whereas only 7 (6%) cases had significantly decreased AA levels which involved aspartic acid, cysteine, glutamic acid and glycine only (Table 1). Interestingly, the maximum number (13) of the 15 measured AAs showing significant increased level occurred at 8h and 24 h time points of the chilling stress, though none of the 15 AAs had significantly increased levels in both genotypes at all time points of the stress except for threonine, isoleucine and valine. Compared to IR29, LTH had more AAs showing significantly increased levels at all time points of the stress (Table 1) and significantly higher levels of cysteine, isoleucine, phe, proline, serine, threonine and valine at 8h of the stress (Additional file 1: Table S1), matching well with its significantly lower levels of cell membrane injury at these time points of the stress (Figure 1).

OA *regulation under chilling stress*: The chilling treatment caused generally decreased levels of most measured OAs in both LTH and IR29 (Table 1; Additional file 1: Table S1). Of the total 248 stress vs control comparisons, 81 (33%) cases had significantly decreased OA levels, whereas only 24 (10%) cases had significantly increased OA levels which involved primarily oxalic acid, isocitric acid, salicylic acid, ascorbic acid and glucopyranuronic acid and occurred primarily in LTH (Table 1). Four OAs (oleic acid, quinic acid, eicosanoic acid and sinapic acid) showed consistently decreased levels across all time points of the stress in both genotypes. Interestingly, four OAs (succinic acid, citric acid, fumaric acid, and malic acid) involved in the tricarboxylic acid cycle (TCA) displayed decreased levels at two or more chilling stress time points. Compared to IR29, LTH had more OAs showing significantly decreased levels at all time points of the stress except at R-24h (Table 1) and significantly lower levels for most OAs at different time points under both stress and control conditions (Additional file 1: Table S1).

*Sugar regulation under chilling stress*: Similar to the measured OAs, most (29) sugars showed decreased levels in response to the chilling treatment in both LTH and IR29 (Table 1; Additional file 1: Table S1). Of the total 304 stress vs control comparisons, 111 (37%) cases had significantly decreased sugar levels which occurred approximately equally in both genotypes. Six sugars (glucopyranose, glucose-6-p, sucrose, lactose, mannitose, trehalose, 3-p-glycerol and sorbitol-6-p) showed consistently reduced levels in both genotypes at all time points of the stress. In 34 (11%) cases, some sugars (benzyl glucopyranoside, galactinol, methyl-beta-d-mannopyranoside, ribose, mannitol and xylitol) showed significantly increased levels which occurred primarily in LTH at the later time points of the stress (Table 1), suggesting that these late-accumulating sugars may be associated with CT of LTH.

### Differential metabolite alteration in LTH and IR29 upon recovery

Upon recovery, 70 (66%) of the measured metabolites were differentially expressed, including 11 AAs, 22 OAs, 24 sugars, 4 polyamines, 4 esters and 5 others (Table 1; Additional file 1: Table S1). At this time point, the difference between LTH and IR29 was at the maximum. Of the 70 differentially regulated metabolites, LTH had significantly increased levels for 37 metabolites (9 AAs, 5 OAs, 13 sugars, 2 polyamines, 3 esters and 4 others) and reduced levels for only 11 ones (6 OAs, 2 sugars, 2 polyamines and 1 ester) (Table 1). In contrast, IR29 had significantly decreased levels for only 12 metabolites (3 AAs, 4 sugars, 2 polyamines, 1 ester and 2 others), but significantly reduced levels for 31 metabolites (1 AA, 18 OAs, 9 sugars, 1 polyamine and 2 others) (Table 1). Those metabolites with significantly increased levels identified specifically in LTH were expected to contribute positively to the better chilling stress recovery of LTH. Under recovery conditions after chilling stress, 9 metabolites (galactose, allantoin, monohexadecanoylglycerol, ribose, triethanolamine, arabinose, isoleucine, cytosine) were commonly increased in both genotypes.

### Correspondence between the metabolomic and transcriptomic responses of LTH and IR29 to chilling stress

**Figure 4:**
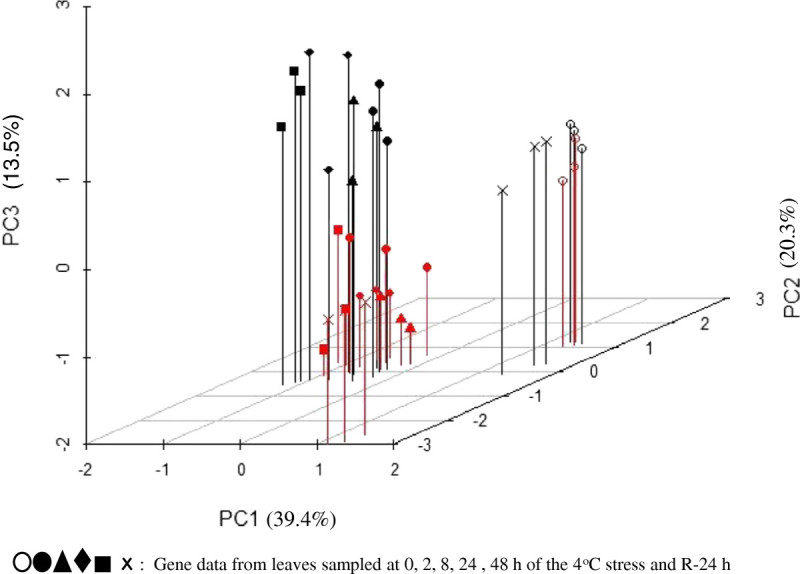
**The plot of the first three principal components of differentially expressed genes (DEGs) involved in the AA, OA and sugar pathways detected in the microarray experiment (Zhang et al.** 2012b**) in two rice genotypes, LTH and IR29, sampled in three biological replicates at the time points of 2, 8, 24, and 48 h after the 4°C chilling stress and 24 h after recovery (R).** The red color and black one indicated IR29 and LTH.

Previously, we discovered 918 differentially expressed genes (DEGs) that are involved in the AA, OA and sugar related pathways in LTH and IR29 under the similar chilling stress (Zhang et al. 2012b). These included 373 DEGs involved in the AA pathways only, 292 DEGs involved in the AA/OA/sugar pathways, 117 DEGs involved in the OA pathways only, 51 DEGs involved in the OA/sugar pathways, and 85 DEGs involved in the sugar pathways only. To uncover the relationships between the metabolomic and transcriptomic responses of LTH and IR29 to the chilling stress, PCA was performed on the log_-10_ transformed DEG data and the results were shown in Figure 4 and Table 2. The PC plot revealed a similar overall picture between the transcriptomic and metabolomic responses (Figure 2) in LTH and IR29. For the DEG data, PC1 accounted for 39.4% of the total variation of the DEG data and separated the chilling (negative side) and control (positive side) treatments on the *X* axis. Of the 918 DEGs, 310 genes had heavy negative loadings on this PC (stress responsive), including 140 (36.5% of the AA DEGs), 94 (32.2% of the AA/OA/sugar DEGs), 39 (37.5% of the OA DEGs), 7 (13.7% of the OA/sugar DEGs) and 30 (35.3% of the sugar DEGs). Also, 438 DEGs had heavy positive loadings (control) on PC1, including 181 (48.5% of the AA DEGs), 143 (49.0% of the AA/OA/sugar DEGs), 53 (45.3% of the OA DEGs), 28 (54.9% of the OA/sugar DEGs), and 33 (38.8% of the sugar DEGs).

**Table 2:** **Comparisons of the PCA results from the differentially expressed metabolites data of 15 amino acids (AAs), 31 organic acids (OAs) and 38 sugars and data of 918 related differentially expressed genes (DEGs) detected in LTH and IR29 under the chilling stress and non-stress control at the seedling stage**

Principal components (PC)	Metabolites or related DEGs^1^	Transcriptomic data		Metabolomic data	
		N	%	N	%
**PC1 (Stress treatments)**	R^2^ (%)		39.4		20.9
	AA^+^	140	37.5	1	6.7
	AA^-^	181	48.5	8	53.3
	(AA+OA+sugar)^+^	94	32.2		
	(AA+OA+sugar^-^	143	49.0		
	OA^+^	39	33.3	25	80.6
	OA^-^	53	45.3	0	0
	(OA+sugar)^+^	7	13.7		
	(OA+sugar)^-^	28	54.9		
	Sugar^+^	30	35.3	29	76.3
	Sugar^-^	33	38.8	0	0
**PC3/PC2 (Genotypic differences)**	R^2^ (%)		13.5		13.8
	AA^+^	118	31.6	8	53.3
	AA^-^	60	19.2	2	13.3
	(AA+OA+sugar)^+^	72	24.7		
	(AA+OA+sugar^-^	66	22.6		
	OA^+^	36	30.8	7	22.6
	OA^-^	29	24.8	17	54.8
	(OA+sugar)^+^	24	47.1		
	(OA+sugar)^-^	7	13.7		
	Sugar^+^	29	34.1	16	42.1
	Sugar^-^	15	17.6	12	31.6
**PC2/PC3 (Genotypic difference in recovery/treatment timing)**	R^2^ (%)	20.3		13.5	
	AA^+^	108	29.0	13	86.7
	AA^-^	102	27.3	0	0
	(AA+OA+sugar)^+^	81	27.7		
	(AA+OA+sugar^-^	97	33.2		
	OA^+^	43	36.8	11	35.5
	OA^-^	35	29.9	5	16.1
	(OA+sugar)^+^	24	47.1		
	(OA+sugar)^-^	16	31.4		
	Sugar^+^	29	34.1	18	47.4
	Sugar^-^	33	38.8	9	23.7

^1^ N is the number of related DEGs or metabolites showing significant positive (^+^) or negative (^-^) loading (contributions) to the corresponding principal components (PCs).

PC2 explained 20.3% of the total variation of the DEG data and separated LTH (positive side) and IR29 (negative side) at R-24 h, in which the DEGs in LTH were largely reversed to the normal status of the non-stress control, whereas those in IR29 remained as in the stress conditions (Figure 4). Thus, this PC summarized the overall different consequences of the chilling stress on LTH and IR29. Of the 918 DEGs, 285 had heavy positive loadings on this PC (quick recovery), including 108 (26.3% of the AA DEGs), 81 (27.7% of the AA/OA/sugar DEGs), 43 (36.8% of OA DEGs), 24 (47.1% of the OA/sugar DEGs) and 29 (34.1% sugar DEGs). Also, 283 DEGs had heady negative loadings (minimum recovery) on PC2, including 102 (26.8% of the AA DEGs), 97 (33.2% of the AA/OA/sugar DEGs), 35 (30% of the OA DEGs), 16 (31.4% of the OA/sugar DEGs), and 33 (38.8% of the sugar DEGs).

PC3 explained 13.5% of the total variation of the DEG data and separated LTH on the positive side and IR29 on the negative side) (Figure 4). Of the 918 DEGs, 279 had heavy positive loadings on this PC (quick recovery of LTH), including 118 (31.6% of the AA DEGs), 72 (24.7% of the AA/OA/sugar DEGs), 36 (30.8% of the OA DEGs), 24 (47.1% of the OA/sugar DEGs) and 29 (34.1% sugar DEGs). Also, 177 DEGs had heady negative loadings (the minimum recovery of IR29) on PC3, including 60 (16.1% of the AA DEGs), 66 (22.6% of the AA/OA/sugar DEGs), 29 (24.8% of the OA DEGs), 7 (17.1% of the OA/sugar DEGs), and 15 (17.6% of the sugar DEGs).

Again, the PCA results revealed 107 important DEGs that had heavy negative loadings on PC1 and positive loading on PC3, which contributed most to the difference in the seedling CT between LTH and IR29. These included 61 of the AA DEGs, 25 of the AA/OA/sugar DEGs, 10 of the OA DEGs, 4 of the OA/sugar DEGs and 7 of the sugar DEGs (Additional file 4: Table S4).

## Discussion

The two genotypes, LTH and IR29, are known to have contrasting CT at the phenotypic and physiological levels (Glaszmann et al. 1990; Baruah et al. 2009). In this study, we further showed that they differed greatly in cell membrane injury caused by chilling stress. The chilling stress treatment of 4°C for two days used in this study were more extreme but resembles the most common low temperature stress rice crops encounter at the seedling stage in northeast China where cold fronts result in a sharp drop in temperature for a couple of days in early Spring. Our experimental design of using stress and control with multiple sampling during a time-course of chilling stress and subsequent recovery was demonstrated to be powerful to quantify the chilling induced dynamic changes of the major groups of primary metabolites in rice, evidenced by the consistent PCA results of the three biological replicates (Figure 2, Additional file 2: Table S2). Our result that levels of most measured metabolites changed significantly in the two rice genotypes in response to the chilling stress clearly indicates that the metabolomic responses of rice to chilling stress involve large numbers of metabolites, which was expected from their huge transcriptomic differences under similar chilling stress (Zhang et al. 2012b). However, considerable variation was observed in expression levels of the same metabolites sampled at different time points of the stress (Additional file 2: Table S2), indicating that multiple samplings and sampling at critical time(s) are essential for studying plant metabolomic responses to abiotic stresses. Nevertheless, our results revealed some interesting aspects in the chilling induced changes of the primary metabolites and their association with seedling CT of rice.

The most important result of this study was that three major groups of the primary metabolites showed distinct change patterns in the two rice genotypes in response to the chilling stress. Firstly, coordinated increases in levels of most AAs occurred in both genotypes at most time points during the chilling stress with the most dramatic increase synchronized the maximum difference in CT between LTH and IR29 during the chilling stress. This strongly suggests that AA accumulation was a general response of rice to chilling stress at the seedling stage. Previously, AA levels have been reportedly to increase in response to different stress treatments in other plants (Kaplan et al. 2004; Nikiforova et al. 2006; Zuther et al. 2007; Armengaud et al. 2009; Araujo et al., 2013; Urano et al. 2009; Bowne et al. 2012), though it remains unclear how the increased AA levels contribute directly or indirectly to plant abiotic stress tolerances. Second, most measured OAs, except for oxalic acid, isocitric acid, salicylic acid, ascorbic acid and glucopyranuronic acid, showed a contrast change pattern to AAs with generally reduced levels in response to the chilling stress in both genotypes, implying that energy production was remarkably inhibited in rice during the chilling stress. While similar results were reported in salt-stressed wheat plants (Wu et al. 2013), our results were somehow contradictory to the expectation that OA levels would increase because of their possible functions as intermediates in plant carbon metabolism and as key components in environmental stress response (López-Bucio et al. 2000; Korn et al. 2010). Third, the chilling induced changes of sugar levels were somewhat between AAs and OAs in which most sugars showed dramatically decreased levels at the earlier time points of the chilling stress and then backed to the normal or higher levels at the later time points of the stress and after the recovery (Table 1). Most sugars have been reported to be positively correlated with freezing tolerance in plants, where they may function as osmoprotectants, nutrients and signaling molecules (Guy et al. 2008; Ma et al. 2009). Increased levels of those soluble sugars have been found in plants under abiotic stresses such as freezing, water deficiency, salinity and low temperature (Hannah et al. 2006; Korn et al. 2010; Guy et al. 2008; Yobi et al. 2012). Finally, our results indicated that the contrast difference in CT between LTH and IR29 were clearly associated with their differentiated metabolomic responses to the chilling stress. While both LTH and IR29 showed the similar change patterns for the three major groups of the primary metabolites, LTH showed more dramatic metabolomic responses to the chilling stress than chilling sensitive IR29, reflected by more metabolites showing chilling induced changes, by greater levels of these metabolomic changes, and by a much greater recovery ability after stress. Detailed examination of those metabolites showing differentiated changes in LTH (Table 1, Additional file 1: Table S1) suggest its high level of seedling CT was at least partially attributed to its increased levels of most AAs and reduced energy consumption that resulted in increased glycolysis and strong resilience on recovery.

The above discussion plus our PCA results clearly indicated that the metabolic pathways involved in producing different groups of metabolites in rice were well coordinated under the chilling stress, suggesting the involvement of plant hormone(s) in their regulation. It appeared that as far as the 106 measured primary metabolites, the chilling induced changes in the levels of many AAs were more the causes of the seedling CT, while the increased levels of specific sugars, OAs and others were more the consequences of rice responses to the chilling stress. These coordinated metabolomic responses were certainly regulated at the transcriptomic level. Indeed, large numbers of genes were differentially expressed in LTH and IR29 under the similar chilling stress and huge differences were observed between LTH and IR29 at the transcriptomic level (Zhang et al. 2012b). Indeed, similar overall variation patterns were observed between the 84 primary metabolites and the 918 related DEGs (Table 2). For example, the maximum variances of both the metabolomic and the transcriptomic data were the same in the direction of the chilling stress treatments (PC1s), and so were the maximum genotypic differences revealed by PC2 of the metabolites data and by the PC3 of the DEG data. However, it was very difficult to find direct and close correspondences between each group of the primary metabolites and their related DEGs, largely because of large groups of DEGs that are involved in pathways leading to different groups of metabolites (Table 2). Similarly, no direct correlation was observed between changes in transcript and metabolite levels under low temperature stress in Arabidopsis (Kaplan et al. 2004). Nevertheless, we did find an interesting and important correspondence between the metabolomic data and trancriptomic data. This was the close correspondence between the seven important AAs (proline, valine, leucine, tryptophan, threonine, serine and tyrosine) that had heavy negative loadings on PC1 (chilling responsive) and positive loadings on PC3 (positive contribution to LTH) and 107 DEGs (86 of which are related to AA pathways) that also had heavy negative loadings on PC1 (chilling responsive) and heavy positive loading on PC3 (positive contribution to LTH). Thus, it remains a huge challenge for future studies to elucidate how this important subset of DEGs are regulated under the chilling stress, that result in corresponding changes in the AAs and other metabolites, and consequently greatly improved CT of LTH.

## Conclusion

GC-MS was used to analyze changes in metabolites of two rice genotypes under chilling stress and subsequent recovery. A genotype- and time-dependent metabolite profile in response to chilling stress was uncovered. Levels of most amino acids increased, whereas most sugars and OAs experienced decreases in both genotypes, demonstrating their differential function against chilling stress. A set of metabolites was exclusively regulated in LTH, indicating their positive roles in chilling tolerance. Many amino acids increased specifically in LTH on recovery, implying that LTH may possess a rapid active renewal mechanism.

## Methods

### Plant growth and chilling stress treatment

Two rice genotypes, LTH (*japonica*) and IR29 (*indica*), were used in this study. LTH and IR29 were previously identified as chilling-tolerant (CT) and chilling-sensitive, respectively (Ye et al. 2008, 2010; Tseng and Teng 1985). Dry seeds of both rice genotypes were sown in plastic plates of 20 × 50 cm filled with rice paddy soil with three plates (three for the chilling treatments and three for the control) as biological replicates. The plates were placed in the growth chambers of the institute of Crop Sciences, Chinese Academy of Agricultural Sciences in Beijing. The rice plants were allowed to grow for 3 weeks at 28°C under a 12/12 h light/dark cycle. When the rice seedlings reached the four-leaf stage, the chilling stress treatments were initiated by exposing the plants to 4°C± 1°C for 48 h, and then transferred to 28°C for 24 h recovery. The control plants were kept at 28°C for the entire growth period.

### *Measurement of* cell membrane injury *under chilling stress and subsequent recovery*

The effect of chilling stress on rice plants was examined by measuring leaf electrolyte leakage, an indicator of cell membrane stability, using the following method (Zhang et al. 2012b). During the 3-day period of the experiment, 0.5 g fresh leaves were sampled from each of the three replicates of the control and treated seedlings at 2 h, 8 h, 24 h, and 48 h after chilling treatment and 24 h after recovery. Leaf samples were cut into 1-cm pieces and immersed in 20 ml distilled water in a tube for 1 h in a vacuum chamber. After standing for 2 h at 25°C, water conductivity was measured as follows: leaf discs were killed in the same solution by autoclaving, and total conductivity was measured at the room temperature. Percent injury arising from each treatment was calculated from conductivity data using the equation: % injury = [(%L_t_ - %L_c_) / (100 - %L_c_)] * 100), where %L_t_ and %L_c_ are percent conductivities for the treated and control samples, respectively.

### Metabolite extraction and their identity determination

The topmost leaves of rice plants of each genotype grown under the control and chilling conditions were harvested at the same five time points as samples for cell membrane injury. All leaf samples were rapidly harvested, flash-frozen in liquid nitrogen (30 s), and stored at -70°C.

For metabolite extraction, frozen leaf samples were ground into powders in liquid nitrogen with a mortar and pestle. Aliquots of frozen powder (100 mg) were extracted based on a modified method used in Bowne et al. (2012). Briefly, 500 μl of 100% methanol was added to the powder samples and incubated at 70°C for 15 min. Samples were centrifuged for 15 min at 14 000 rpm and the supernatant transferred to a new tube, then 500 μl of water was added and 30 μl aliquoted to new tubes for TMS derivatization. Ribitol was added as an internal standard. A C12, C15, C19, C22, C28, C32, and C36 n-alkane mixture was used for determining the retention time indices (RIs). The extracted metabolite samples were derivatized as described by Bowne et al. (2012), and analyzed using an MD 800 GC-MS system (ThermoQuest, Manchester, UK). The leaf mixture of both genotypes under both the stress and control conditions was extracted in bulk as the reference samples, and one reference sample was run every ten samples. Meanwhile, the *N*-methyl-*N*-[trimethylsilyl] trifluoroacetamide (MSTFA) was run once every five samples to clean the potential pollution on the injection surface.

Chromatograms and mass spectra were processed using the find algorithm in the MassLab version 1.4 software (ThermoQuest). Specific mass spectral fragments were detected in defined retention time windows using the mass spectral library NIST (http://www.nist.gov/mml/chemical_properties/data/electionlibcomp.cfm) and the public domain mass spectra library of the Max-Planck-Institute for Plant Physiology, Golm, Germany (http://csbdb.mpimp-golm.mpg.de/csbdb/gmd/msri/gmd_msri.html). In order to detect as more metabolites as possible, each derivatized sample was run two times with split ratios of 1:10 and 1:2, respectively. The metabolite identity was determined by comparing the mass spectra similarity of the samples to that in the mass library with match level ≥ 75% and RI deviation ≤5. The quantification of metabolite was based on the peak area. Further confirmation of most identified AAs, OAs, and sugars was performed via the standard addition experiments using the pure authenticated compounds. Totally 106 metabolites were identified at the present study (Additional file 5: Table S5).

### Data analysis

The data normalization was performed as described by (Bowne et al. 2012). The denominator of the quotient was the average response of the reference samples. The sample responses were volume-corrected with ribitol for error during the sample preparation or GC injection and normalized using sample fresh weight.

Analyses of variance (ANOVA) was performed to determine the significances of the differences between the genotypes (G), between the chilling treatments (T), among the sampling time points within the treatment (T_(t)_), and their interactions using SAS Proc GLM (SAS Institute Inc. 1996). Differentially changed metabolites were defined as those showing significant concentration increases or decreases relative to their respective controls at P ≤ 0.05 in ANOVA. Because the measured metabolites of each major group (AAs, OAs and sugars, etc.) are produced in related pathways and thus expected to show correlated responses to the chilling stress, a principal component analysis (PCA) was performed on log_10_-transformed relative responses of the metabolite data using the SPSS software to understand the co-variance structure of the measured metabolite data and to reveal the overall variation pattern of the metabolomic responses of the two rice genotypes to the chilling stress. In addition, a comparative PCA was performed using log_10_ transformed data from 918 differentially expressed genes with known functions in pathways of AAs, OAs and sugars identified previously in LTH and IR29 under virtually the same chilling stress treatments (Zhang et al. 2012b).

## Electronic supplementary material

Additional file 1: The mean values of 106 primary metabolites from leaves of IR29 and LTH measured at 2 h, 8 h, 24 h and 48 h of the 4°C chilling treatment and non-stress control conditions, and upon 24 h recovery after the treatment. (XLS 94 KB)

Additional file 2: ANOVA results for 106 metabolites from leaves of two rice genotypes (G), IR29 and LTH, measured at time points (t) of 2 h, 8 h, 24 h and 48 h of the 4°C chilling treatment and and 24 h recovery after and the non-stress control (T). (XLS 72 KB)

Additional file 3: The loadings (contributions) of each of the 106 metabolites to the first three principal components (PC). (XLS 36 KB)

Additional file 4: Loadings of 918 differentially expressed genes (DEGs) on the first 3 principal components of the PCA. (XLS 202 KB)

Additional file 5: The conditions for determining and confirming the identities of the measured metabolites. (XLS 38 KB)

Below are the links to the authors’ original submitted files for images.

Authors’ original file for figure 1

Authors’ original file for figure 2

Authors’ original file for figure 3

Authors’ original file for figure 4
