# Supplementary figures and images for: Temporal profiling of primary metabolites under chilling stress and its association with seedling chilling tolerance of rice (Oryza sativa L.)
Source: Rice (N Y). 2013 Oct 5;6:23. doi: 10.1186/1939-8433-6-23 (PMC4883686; doi:10.1186/1939-8433-6-23)

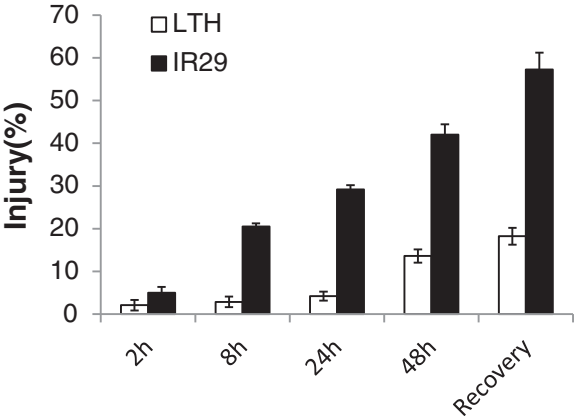

Supplement: Supplementary file 6 — Authors’ original file for figure 1 [file 12284_2013_59_MOESM6_ESM.pdf]

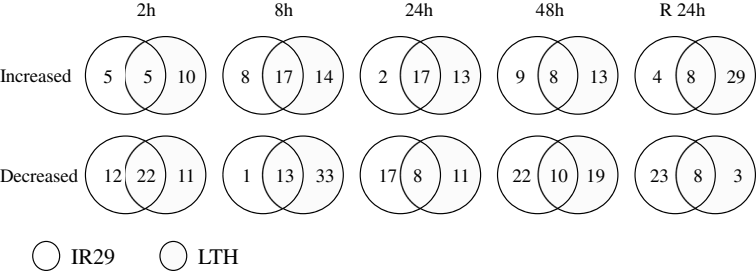

Supplement: Supplementary file 8 — Authors’ original file for figure 3 [file 12284_2013_59_MOESM8_ESM.pdf]

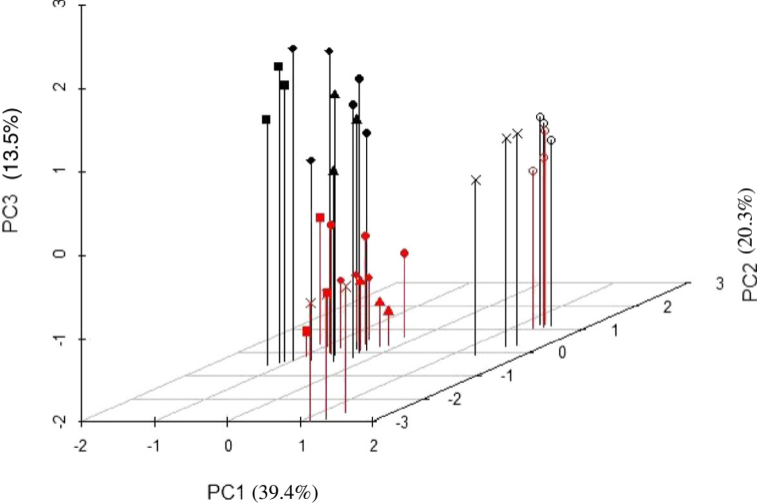

○●▲◆■ X : Gene data from leaves sampled at 0, 2, 8, 24, 48 h of the 4°C stress and R-24 h

Supplement: Supplementary file 9 — Authors’ original file for figure 4 [file 12284_2013_59_MOESM9_ESM.pdf]
