# Supplementary material for: Temporal profiling of primary metabolites under chilling stress and its association with seedling chilling tolerance of rice (Oryza sativa L.)
Source: Rice (N Y). 2013 Oct 5;6:23. doi: 10.1186/1939-8433-6-23 (PMC4883686; doi:10.1186/1939-8433-6-23)
Supplement: Supplementary file 7 — Authors’ original file for figure 2 [file 12284_2013_59_MOESM7_ESM.pdf]

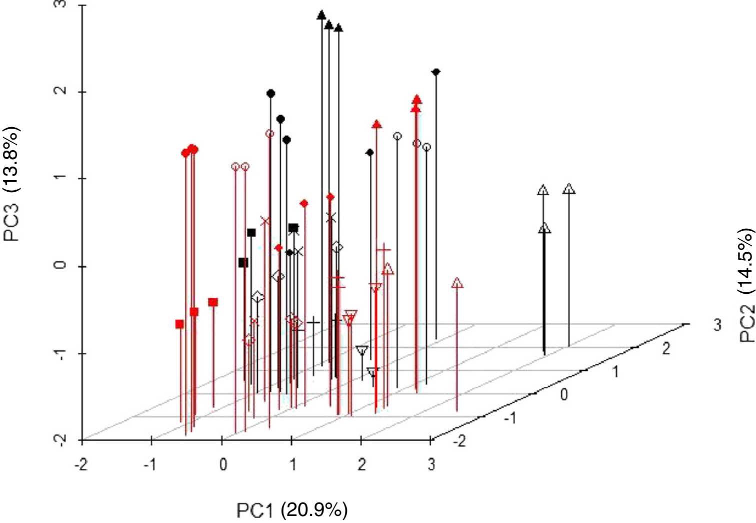

○ △ ▽ ◇ + : Metabolite data from leaves sampled at 2, 8, 24, 48 h and R-24 h under the non-stress control

● ▲ ◆ ■ × : Metabolite data from leaves sampled at 2, 8, 24 , 48 h of the 4°C stress and R-24 h
